# Comparison of Fluorescence *In Situ* Hybridization and Chromogenic *In Situ* Hybridization for Low and High Throughput *HER2* Genetic Testing

**DOI:** 10.1155/2013/368731

**Published:** 2013-12-05

**Authors:** Tim S. Poulsen, Maiken L. M. Espersen, Vibeke Kofoed, Tanja Dabetic, Estrid Høgdall, Eva Balslev

**Affiliations:** Molecular Unit, Department of Pathology, Herlev Hospital, University of Copenhagen, Herlev Ringvej 75, 2730 Herlev, Denmark

## Abstract

The purpose was to evaluate and compare 5 different *HER2* genetic assays with different characteristics that could affect the performance to analyze the human epidermal growth factor 2 (*HER2*) gene copy number under low and high throughput conditions. The study included 108 tissue samples from breast cancer patients with HER2 immunohistochemistry (IHC) results scored as 0/1+, 2+, and 3+. *HER2* genetic status was analysed using chromogenic *in situ* hybridization (CISH) and fluorescence *in situ* hybridization (FISH). Scoring results were documented through digital image analysis. The cancer region of interest was identified from a serial H&E stained slide following tissue cores were transferred to a tissue microarrays (TMA). When using TMA in a routine flow, all patients will be tested for HER2 status with IHC followed by CISH or FISH, thereby providing individual *HER2* results. In conclusion, our results show that the differences between the *HER2* genetic assays do not have an effect on the analytic performance and the CISH technology is superior to high throughput *HER2* genetic testing due to scanning speed, while the IQ-FISH may still be a choice for fast low throughput *HER2* genetic testing.

## 1. Introduction

Human Epidermal growth factor Receptor 2 (HER2) expression is investigated routinely on all breast cancer cases to make the therapeutic decisions for patients with breast cancer. The American Society of Clinical Oncology (ASCO)/College of American Pathologist (CAP) recommendations for HER2 status testing are first immunohistochemical (IHC) staining and secondary to perform genetic *HER2* testing on tissues scored as borderline cases (2+) found by IHC [1]. Ratio-based dual color *HER2* gene amplification assays are commercially available from a multiple vendors using either fluorescence *in situ* hybridization (FISH) or chromogenic *in situ* hybridization (CISH), where the various tests have differing characteristics (Table 1). The *HER2*/neu labeled part of the dual color *HER2* genetic assays is in all cases DNA based, while the centromere reference part can either be an DNA probe or an peptide nucleic acids (PNA) probe. The direct labeling of the different FISH probes from Dako and ZytoVision uses red (TexasRed), orange (Rhodamine), or green (FITC) fluorocrome, while the CISH-based assays give rise to either red, green, or blue chromogenic precipitation. Various strategies for blocking of nonspecific probe binding and detection systems have been implemented into the different *HER2* genetic assays. ZytoVison uses repeat-free oligonucleotides and thereby does not need to block repeated sequences (e.g., alu, LINE, and SINE) while another system (Dako) has developed alu sequence blocking peptide nucleic acids (PNAs) to lower the background generated from the repeated sequences that are located in the *HER2* DNA probe [2, 3]. A newly developed hybridization technique *HER2* IQ-FISH (Dako) reduces the assay time from two days to four hours. This is achieved by breaking the hydrophobic forces in the DNA helix used in stacking [4] with the polar aprotic ethylene carbonate instead of attacking the hydrophilic hydrogen bonds between the bases that are normally broken with the use of form amide. Destabilization of the DNA helix with a polar aprotic solvent results in faster reannealing of the internal genomic repetitive sequences, thereby preventing the need for blocking of repeated sequences in the IQ-FISH assay. FISH has been regarded as the gold standard for *HER2* gene copy number determination in breast cancer [5]. CISH has been introduced to simplify the evaluation of the gene signals and to be able to compare the tumour area with H&E stained slides using a conventional bright field light microscope [6]. Multiple studies have shown that tissue microarray (TMA) can be used for *HER2* genetic testing and still has a high sensitivity and specificity during routine diagnostic of breast cancer [7–11]. The aims of our study were to examine the robustness of 5 different genetic *HER2* assays in a high throughput routine setting using TMA containing breast cancer tissue and to evaluate if different characteristics between the five *HER2* genetic assays could affect the perfomance when using digitalization of the *HER2* stained slides before manually scoring on a monitor screen.

## 2. Materials and Methods

### 2.1. Patients

The study included 108 consecutive breast carcinomas from patients diagnosed at Herlev Hospital, Denmark, with information of HER2 status performed by IHC.

### 2.2. Tissue Microarray Construction (TMA)

TMAs were constructed from formalin-fixed and paraffin-embedded donor blocks, using a fully automated ATA-27 (Beecher Instrument). The areas of interest at the margin of tumor were marked on H&E stained slides by a pathologist. Four cores of 1 mm or one core of 2 mm in diameters was used per donor block and mounted in a new recipient block. Tissue preparation, microscopy, and subsequent laboratory analyses were carried out as part of the daily routine.

### 2.3. Chromogenic (CISH) and Fluorescent (FISH) *In Situ* Hybridization

The *HER2* genetic testing was performed using 5 assays: *HER2* CISH pharmDx Kit-SK109, *HER2* FISH pharmDx Kit-K5331, *HER2* IQ-FISH pharmDx-K5731 (Dako, Denmark), ZytoDot 2C SPEC *HER2*/CEN17 Probe Kit-C-3022-40 and SPEC *HER2*/CEN17 Dual Colour Probe Kit-Z-2020 (ZytoVision, Germany). The tests were conducted according to the manufacturers' instructions with minor changes, which are specified below. All samples were treated with pepsin for 8 minutes at room temperature. The last step of the ZytoDot CISH dehydration (3x 30 s in 100% ethanol and incubate 2x 30 s in xylene) was changed to air drying for 30 minutes before mounting. This preserves the red signals which can be faintly stained when using xylene or ethanol and avoids trapping of bubbles underneath the coverslip caused by water or air.

### 2.4. Digitalizing and Scoring

The CISH- and FISH-stained TMAs were scanned using a bright field/fluorescent panoramic scan (3D HisTech) equipped with a 40x dry objective and using single focus layer for CISH TMAs and five focus layers separated by 0.75 microns (z-stacking) for FISH TMAs. The scanned TMA full slide was analysed using Panoramic Viewer and manually scored on a computer monitor. Scores for *HER2* genetic testing were counted without knowledge of patient outcome, IHC status and results from other *HER2* genetic testing. Three separate tumor areas were selected and at least 60 signals (either red or green) from invasive tumor cells were counted [12]. All overlapping nuclei were excluded; only nuclei with a distinct nuclear border were being evaluated. The score was reported as the ratio between *HER2* gene and centromere 17 (CEN17). Scoring criteria used for analysis were nonamplified (<1.8), equivocal (1.8–2.2), and amplified (>2.2) according to ASCO/CAP guidelines [1, 13]. For all equivocal *HER2* ratios, another 60 signals were scored and the final ratio of the case was calculated from the total number of signals. The final scoring of the reanalysed equivocal *HER2* ratios was reported according to the cut-off criteria nonamplified (<2.0) and amplified (≥2.0).

### 2.5. Statistic

The statistical analysis of the accuracy was performed by calculating the agreement between a constructed consensus assay from the five different *HER2* genetic assays and *κ* statistics [14].

## 3. Results

A total of 108 breast carcinomas were included in the study and 5 different *HER2* genetic assays (FISH *n* = 3, CISH *n* = 2) were investigated, resulting in 540 scoring results. The success rate of FISH and CISH *HER2* genetic testing during routine condition was 100%. The scanning success was 97,6% (527 out of 540). Thirteen samples failed; of those FISH accounted for 11 samples and CISH accounted for 2 samples. Failures of FISH scanning were missing autofocus, high background staining, and persistent autofluorescence. Failures of CISH scanning for the two samples were caused by a fingerprint and an air bobble captured underneath the coverslip. These 13 patients were excluded (*n* = 65 scoring results), resulting in a total cohort of evaluated patients of 95 (*n* = 475 scoring results). All thirteen scanning failures were located on the 2 mm core TMA's, while at least two of the four cores on the 1 mm core TMA could be scanned and analysed successfully. The mean digital imaging scanning time of CISH using a 40x objective was 29 sec per mm^2^, while the FISH stained slides using five extended focus layers for the *HER2* and CEN17 filters and a single layer for DAPI were 764 sec/mm^2^.

When using the mean *HER2* ratio of CISH and FISH scores, concordance was found in 99% (94/95) of the cases (Cohen *κ* coefficient, 0,9664), which support earlier published results [12, 14, 15] (Table 2). One tissue core was scored as nonamplified by CISH (ratio = 1.9) and amplified by FISH (ratio = 2.3). We observed high concordance between the different FISH and CISH assays when a single assay was compared to a consensus generated scoring of the remaining four *HER2* genetic assays. The concordance within the CISH assays was 97.9% and 99.0% (Tables 3(a) and 3(b)), while the concordance within the FISH assays were 97.9%, 97.9%, and 99.0% (Tables 3(c), 3(d), and 3(e)), respectively.

Discordant *HER2* ratio scoring results of the FISH and CISH assays were found in 6% of the patients (Table 4). In case 15 three assays showed nonamplification while two assays showed amplified *HER2*/CEN17 ratio. The scorings were very close to the borderline indicated by four of the assays while one (FISH, Dako) showed 3.0 *HER2*/CEN17 ratio amplification. An explanation for this difference is that it may be caused by tumour heterogeneous amplification, as a region on the 2 mm core were clearly *HER2* amplified. Case 69, four assays showed amplification and one FISH assay showed nonamplification. The divergent scoring results may be due to tumour heterogeneous amplification, where the correct amplified tumour area was not identified on the 2 mm core. With cases 48 and 84, the scorings were very close to the borderline indicated by *HER2*/CEN17 ratio amplification close to 2.0. The IHC scores were 2+ and 1+, respectively (Table 4). This indicates that true borderline cases cannot be solved either by FISH, CISH, or IHC. Case 74: four assays showed non-amplification and one FISH assay showed amplification. The faint green signals from the CEN17 probe may be the reason as it causes an underestimation of the amount of signals. Case 90: four assays showed amplification and one CISH assay showed non-amplification. The finding is due to cluster amplification where the red chromogenic staining gave a purple colour and thereby could be misread as a blue colour.

## 4. Discussion

HER2 testing is required to identify of breast cancer patients that may benefit for trastuzumab adjuvant therapy. Significant correlation in *HER2* status between dual-colour CISH and FISH analysis is reported as well as a reduction in scoring time and laboratory hands on time [7, 9, 16–18]. The mean dual-colour CISH *HER2* copy number and the mean *HER2*/CEN17 ratio were lower than those estimated with FISH but did not result in discrepancy of the final result. The number of *HER2* counted signals can be underestimated due to overlapping signals. This discrepancy is only a risk in low amplified cases and in our study it was only seen in heavily amplified *HER2* signals.

Introduction of TMA into a clinical routine laboratory can be a useful technique when a high throughput of *HER2* analyses is needed. By using TMA, an additional possibility emerges to analyse all patients with regard to both IHC HER2 and *HER2* genetic status without considerable increase of cost [7].

In most tumours, the *HER2*/CEN17 is homogeneously amplified; however in approximately 10% of the carcinomas, *HER2* genetic testing shows unusual signal pattern [19, 20]. We identified three different *HER2* amplification staining patterns. Homogeneously amplified tumors with identical results in scored areas, intratumour heterogeneous amplification/nonamplification areas, in which a minimum of one region was scored as amplified and another scored nonamplified and can be considered as hot spot. A third heterogeneous amplification pattern was illustrated by a single cell with extensive amplification surrounded by cells that are nonamplified. However, little is known about the clinical implication of such patterns, except for one study demonstrating intratumour heterogeneity of *HER2* gene amplification to be associated with a shorter disease-free survival [20]. We used 4 cores from different areas in the periphery of the carcinoma and found that all cores should be included in the counting of dots to get a correct result.

The different use of DNA and PNA probes together with different blocking reagents against repeated regions did not affect the genetic test results.

To our knowledge, the first study to analyse the difference between CISH and FISH for high throughput *HER2* genetic testing aimed at identifying the best technique for preparation of digital imaging and scanning. The high level of agreement obtained between CISH and FISH genetic testing with respect to assay performance makes the two techniques equivalent, in a technical perspective. The most important advantage of CISH over FISH is the much faster digitalization of a CISH-stained slide in combination with low failure rate. The success of CISH digitalization is an advantage for a future automatic image analysis of *HER2* genetic testing in a routine laboratory.

## 5. Conclusion

In conclusion, our results show that the differences between the five *HER2* genetic assays do not have effect on the analytic performance and the CISH technology is superior to high throughput *HER2* genetic testing due to scanning speed, while the IQ-FISH may still be a choice for fast low throughput *HER2* genetic testing when only analysis of a few patients is required.

## Figures and Tables

**Table 1:** Different characteristics between the five **HER2** genetic assays.

	Dako *HER2* FISH		Dako *HER2* IQ-FISH		Dako *HER2* CISH		ZytoVision *HER2* FISH		ZytoVision *HER2* CISH	
Gene	*HER2*	CEN17	*HER2*	CEN17	*HER2*	CEN17	*HER2*	CEN17	*HER2*	CEN17
Probe	DNA	PNA	DNA	PNA	DNA	PNA	DNA	DNA	DNA	DNA
Label color	TxRed	FITC	TxRed	FITC	Red	Blue	FITC	Rhodamine	Green	Red
Blocking reagent	alu-PNA		alu-PNA		alu-PNA		Repeat free		Repeat free	
Visualization	Fluorescence		Fluorescence		Chromogenic		Fluorescence		Chromogenic	
Hybridization reagent	Formamide		Ethylene carbonate		Formamide		Formamide		Formamide	

CISH: chromogenic *in situ* hybridization; FISH: fluorescence *in situ* hybridization; PNA: peptide nucleic acids.

**Table 2:** Performance of mean CISH and mean FISH *HER2* ratio assay.

CISH	FISH		
	Amplified (≥2.0)	Nonamplified	Total
Amplified (≥2.0)	18	0	**18**
Nonamplified	1	76	**77**
Total	**19**	**76**	**95**

CISH: chromogenic *in situ* hybridization; FISH: fluorescence *in situ* hybridization; CI: confidence interval. Concordance, 99,0%; Cohen *κ* coefficient, 0,9664 (95% CI, 0,9010–1,0000).

**Table:** (a)

Consensus (mean of the other four assays)	CISH ZytoVision		
	Amplified (≥2.0)	Nonamplified	Total
Amplified (≥2.0)	18	1	**19**
Nonamplified	1	75	**76**
Total	**19**	**76**	**95**

Concordance, 97,9%; Cohen *κ* coefficient, 0,9342.

**Table:** (b)

Consensus (mean of the other four assays)	CISH Dako		
	Amplified (≥2.0)	Nonamplified	Total
Amplified (≥2.0)	18	1	**19**
Nonamplified	0	76	**76**
Total	**18**	**77**	**95**

Concordance, 99,0%; Cohen *κ* coefficient, 0,9664.

**Table:** (c)

Consensus (mean of the other four assays)	FISH ZytoVision		
	Amplified (≥2.0)	Nonamplified	Total
Amplified (≥2.0)	18	1	**18**
Nonamplified	1	76	**77**
Total	**19**	**76**	**95**

Concordance, 97,8%; Cohen *κ* coefficient, 0,9664.

**Table:** (d)

Consensus (mean of the other four assays)	FISH Dako		
	Amplified (≥2.0)	Nonamplified	Total
Amplified (≥2.0)	18	0	**18**
Nonamplified	2	75	**77**
Total	**20**	**75**	**95**

Concordance, 97,9%; Cohen *κ* coefficient, 0,9343.

**Table:** (e)

Consensus (mean of the other four assays)	IQ-FISH Dako		
	Amplified (≥2.0)	Nonamplified	Total
Amplified (≥2.0)	17	1	**18**
Nonamplified	0	77	**77**
Total	**17**	**78**	**95**

Concordance, 99,0%; Cohen *κ* coefficient 0,9650.

CISH: chromogenic *in  situ* hybridization; FISH: fluorescence *in situ* hybridization.

**Table 4:** Discrepancy cases between the five different *HER2* genetic ratio assays.

	CISH ZytoVision	CISH Dako	FISH ZytoVision	FISH Dako	IQ-FISH Dako	IHC HER2
Case 15	1,7	**2,1**	1,9	**3,0**	1,9	2+
Case 48	2,1	2,1	2,1	2,3	**1,9**	2+
Case 69	3,7	4,8	7,1	7,2	**1,4**	2+
Case 74	1,5	1,2	1,6	**2,2**	1,6	1+
Case 84	**2,0**	1,4	**2,0**	1,9	1,8	1+
Case 90	2,9	**1,9**	2,4	2,4	3,7	2+

CISH: chromogenic *in situ* hybridization; FISH: fluorescence *in situ* hybridization.
